# Metastatic Prostate Adenocarcinoma Presenting Central Diabetes Insipidus

**DOI:** 10.1155/2012/452149

**Published:** 2012-03-18

**Authors:** Hakkı Yılmaz, Mustafa Kaya, Mücteba Can, Mustafa Özbek, Bahir Keyik

**Affiliations:** ^1^Department of Endocrinology and Metabolism, Dışkapı Yıldırım Beyazıt Education and Researching Hospital, Altındağ, 06110 Ankara, Turkey; ^2^Department of Internal Medicine, Dışkapı Yıldırım Beyazıt Education and Researching Hospital, Altındağ, 06110 Ankara, Turkey; ^3^Department of Radiology, Dışkapı Yıldırım Beyazıt Education and Researching Hospital, Altındağ, 06110 Ankara, Turkey

## Abstract

The pituitary gland and infundibulum can be involved in a variety of medical conditions, including infiltrative diseases, fungal infections, tuberculosis, and primary and metastatic tumors. Metastases to the pituitary gland are absolutely rare, and they are generally secondary to pulmonary carcinoma in men and breast carcinoma in women. Pituitary metastases more commonly affect the posterior lobe and the infundibulum than the anterior lobe. The posterior lobe involvement may explain why patients with pituitary metastases frequently present with diabetes insipidus. We are presenting a case report of a 78-year-old male patient who had metastatic prostate with sudden onset of polyuria and persistent thirst. He had no electrolyte imbalance except mild hypernatremia. The MRI scan of the brain yielded a suspicious area in pituitary gland. A pituitary stalk metastasis was found on magnetic resonance imaging (MRI) of pituitary. Water deprivation test was compatible with DI. A clinical response to nasal vasopressin was achieved and laboratory results revealed central diabetes insipidus. As a result, the intrasellar and suprasellar masses decreased in size, and urinary output accordingly decreased.

## 1. Introduction

Diabetes insipidus is a disorder of a large volume of urine (diabetes) that is hypotonic, dilute, and tasteless (insipid). This is opposed to the hypertonic and sweet urine of diabetes mellitus (honey). Four pathophysiologic mechanisms related to vasopressin produce large volumes of dilute urine and polydipsia:

1. hypothalamic (central or neurohypophyseal) diabetes insipidus with inability to secrete and usually to synthesize vasopressin in the neurohypophyseal system,
2. nephrogenic diabetes insipidus wherein there is an inappropriate renal response to vasopressin,
3. transient diabetes insipidus of pregnancy produced by the accelerated metabolism of vasopressin,
4. primary polydipsia wherein the initial pathophysiology is the ingestion of fluid rather than the excretion of fluid.

Metastatic disease involving the pituitary is usually found in association with widespread metastatic disease and may only be reported at autopsy, being asymptomatic during life. Metastases are twice as likely to involve the posterior pituitary as the anterior pituitary, which is thought to be due to a more direct arterial blood supply to the posterior pituitary. Most primary tumors in the hypothalamic-pituitary area that cause diabetes insipidus are relatively slow growing, and any tumor in this area that shows rapid growth in a short period of time should be considered to be a possible metastatic tumor.

 Diabetes insipidus is reported with lymphomas in the hypothalamic/pituitary area. There may be some increased incidence of lymphoma presenting with diabetes insipidus due to the increased incidence of lymphoproliferative disease with human immunodeficiency virus (HIV) and hepatitis C infection. Diabetes insipidus is also associated with leukemia. The mechanism is thought to be infiltration of the hypothalamus, thrombosis, or infection. Diabetes insipidus is distinctly more common in nonlymphocytic leukemia. MRI studies in leukemia may show infiltration or an infundibular mass, but often results are normal even when leukemic cells are found in the cerebrospinal fluid (CSF).

Primary or secondary (most often due to lung cancer, leukemia, or lymphoma) tumors in the brain can involve the hypothalamic-pituitary region and lead to central diabetes insipidus (CDI). In some patients with metastatic disease, polyuria is the presenting symptom. We found two case reports about pituitary gland metastasis from carcinoma of the prostate [1, 2]. But two case reports presented pituitary mass affect no central diabetes insipidus. We reported the first case about apatient who has central diabetes insipidus with metastatic prostate cancer due to only posterior pituitary metastasis.

## 2. Case Report

**Figure 1:**
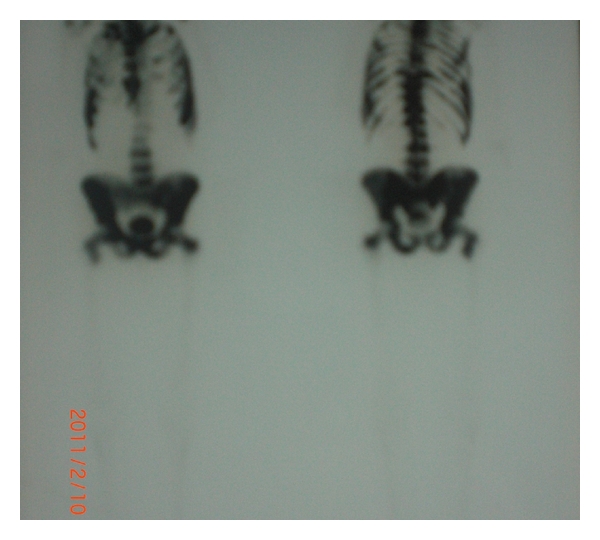
Bone scan: all bone metastases—rib, femur, pelvis, spine bone metastases.

**Figure 2:**
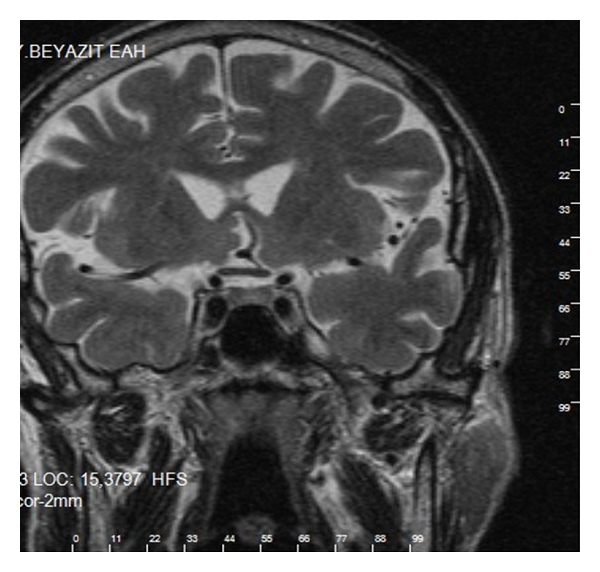
T2-weighted MRI demonstrated that 5 × 8 mm mass the has irregular borderline.

**Figure 3:**
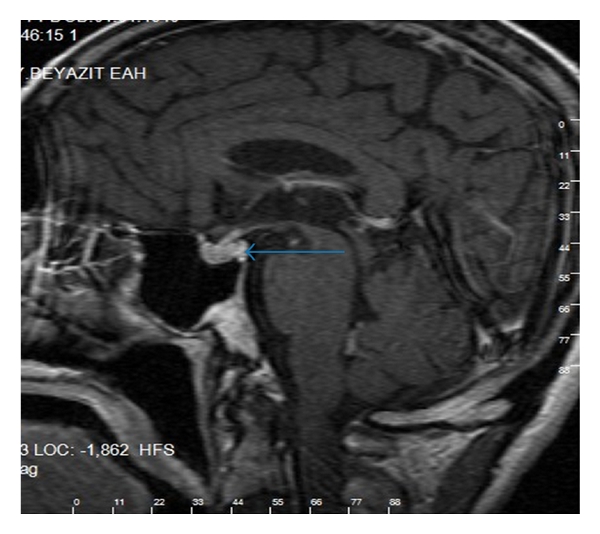
MRI showed that 5 × 8 mm mass the has irregular borderline (arrow).

A man 76-year-old was admitted to our hospital with urinary urgency. On admission, the other systems examinations were normal. But on digital rectal examination, there was asymmetric areas of induration. First PSA: 18.2 ng/mL. Transrectal biopsy of the prostate was performed with transrectal ultrasound (TRUS)-guided. Biopsy has proven Geason grade 4 + 4 = score 8 adenocarcinoma. He was treated with open prostatectomy, and TNM stage is T3 N0 M0. Hormonal treatment with leuprolide was started. He has used the drug presently. One year later, he complained back pain. He did not come back to my hospital, but six months later, he complained about polyuria, polydipsia, and loss of weight without blindness, ophthalmoplegia in his eyes, and impairment of memory and mental status. Other problems were constipation, loss of appetite, and drowsiness. He did not have a headache. Physical examination showed signs of dehydration. PSA level was 120 ng/mL (normal < 4 ng/dL). Further laboratory results are shown in Table 1. A 99mTc-diphosphate bone scan revealed metastases in the spine, ribs, and femurs (Figure 1). MRI-scan with gadolinium contrast suggested a lesion (5 × 8 mm) in the posterior part of the pituitary, relatively isodense to the brain in T1- and T2-weighed images, and loss of the normal high signal from the posterior lobe of the pituitary gland (Figures 2 and 3). We think central diabetes insipidus secondary to metastatic prostate carcinoma. We began desmopressin therapy (10 microg of the nasal spray) for central diabetes insipidus. We suggested local external beam radiation therapy for lumbar and thoracic vertebral bones and pituitary with chemotherapy to the patient, but he did not accept. He improved symptoms (polyuria, polydipsia); Sodium (from 151 to 137), and urine output decreased (from 9.2 to 2.1) two weeks later in Table 1.

## 3. Discussion

Diabetes insipidus is a syndrome characterised by hypotonic polyuria and polydipsia, either as a result of inadequate antidiuretic hormone (ADH) secretion central diabetes insipidus (CDI), inadequate renal response to ADH (nephrogenic diabetes insipidus), or primary polydipsia [3, 4]. Causes of CDI are congenital or acquired lesions that disrupt the neurons that originate in the supraoptic and paraventricular nuclei of the hypothalamus axis. These lesions are malformations, damage resulting from surgery or trauma, tumours, haemorrhage, thrombosis, infarction or granulomatous disease. Some 30 to 50% of cases are idiopathic [5, 6]. Primary tumours are craniopharyngioma, meningioma, or germinoma, but secondary tumours can also occur. Metastases in the posterior pituitary lobe are more common than in the anterior lobe, possibly caused by the direct blood supply of the posterior lobe from the systemic circulation. In 1857, L. Benjamin first described a case of metastasis to the pituitary (MP) gland in an autopsy of a patient with disseminated melanoma [5]. Since then, a number of surgical and autopsy series and sporadic case reports have described metastatic tumors to the pituitary. MP is an infrequent clinical problem reported in 0.14–28.1% of all brain metastases in autopsy series [5–7].

 Breast cancer is the most common tumor to metastasize to the pituitary gland (varying from 5.3 to 28%) [8, 9]; its frequency is followed by that of lung cancer. Prostate [1, 2], renal cell [10, 11], gastrointestinal cancers [12], lymphoma [13], leukemia, thyroid carcinoma [14], and plasmacytoma [15] have also been reported. We hypothesized that the posterior pituitary, by receiving a direct arterial blood supply, is more likely to develop metastases than the adenohypophysis, which receives its blood supply from the hypophyseal portal system. Therefore, hematogenous spreads of malignant cells disseminate easier to posterior part of hypophysis than to the anterior lobe, which is supplied by hypophyseal portal system.

 MRI is the most useful imaging modality for pituitary pathologies. Hypophysis, stalk, cavernous sinuses, sphenoid sinus, and optic chiasma are well evaluated on coronal and sagittal T1-weighted MRI both before and after Gadolinium injection [16]. Lesions on MRI may be seen as cystic, nodular, or stalk thickening. On MRI, the posterior pituitary is identified by hyperintensity, probably caused by phospholipids or secretory granules in pituicytes [17]. A lack of this hyperintensity on sagittal T1-weighted images, as was observed in our patient, is the hallmark of hypothalamic posterior pituitary disorders and may represent an early stage of tumour infiltration [4, 18]. A thickened pituitary stalk could be another indicative finding [19]. We found two case reports about pituitary gland metastasis from carcinoma of the prostate [1, 2]. But two case reports presented pituitary mass affect no central diabetes insipidus. We reported a first case of a patient who has central diabetes insipidus with metastatic prostate cancer due to only posterior pituitary metastasis.

 Management of these patients may also be very difficult because the prognosis depends on the course of the primary neoplasm. Treatment, being basically palliative, depends on the symptoms and the extent of the systemic disease [20]. In known cancer patients, a full investigation for additional metastases is mandatory. Multiple treatment modalities exist for pituitary metastases (MP) including resection, radiation therapy, and chemotherapy. The surgical indications are a symptomatic mass lesion [20]. Nonetheless, surgery is more difficult compared to primary pituitary adenomas because blood loss is greater, and the lesion could be attached to the surrounding structures or show considerable invasiveness [20]. Reports of surgery of MPs indicate that the lesions tend to be firm, diffuse, invasive, vascular, and hemorrhagic; therefore, their total resection is unlikely. In this setting, local radiation and/or chemotherapy are recommended as the initial course of treatment, especially in patients with widespread metastases, in combination with pituitary hormone substitution therapy. Surgical exploration and decompression, alone or combined with radiation, is essential if clarification of diagnosis is likely to affect therapy or if suprasellar extension causes progressive deterioration in vision or pain. Surgery and radiation are well tolerated in noncompromised patients, being associated with low morbidity and minimal complications. Radiosurgery (gamma knife radiosurgery) may be considered less invasive than conventional radiation therapy from the standpoint of radiation effects on the surrounding structures. One patient has been treated by gamma knife radiosurgery with metastatic pituitary carcinoid tumor resulting in rapid decrease of tumour size [21].

## 4. Conclusion

In conclusion, in cancer patients who have symptoms such as nausea, vomiting, polyuria, and polydipsia while they are not on chemotherapy should be evaluated for not only metabolic complications like hypercalcemia and hypokalemia but also posterior pituitary or stalk metastasis. MRI could be the choice of imaging for pituitary metastasis. We could think that sudden onset of diabetes insipidus pituitary metastases should be taken in to account in differential diagnosis.

## Figures and Tables

**Table 1:** Baseline and after treatment endocrinological and biochemical evaluation.

	First	After treatment	Normal range
Serum			
TSH	0.60	0.85	0.4–5.5 microIU/mL
Free T4	1.28	1.31	0.74–1.52 ng/dL
Free T3	2.4	2.6	2.3–4.2 pg/mL
FSH	5.3	4.1	1.9–18.9 IU/L
LH	0.7	0.8	1.7–9.6 IU/L
Total testosterone	18	14	370–1000 ng/dL
Prolactin	5.8	12.1	2.7–18.3 ng/mL
IGF-1	98	100	84–238 ng/mL
ACTH	14.2	15.1	7–46 pg/mL
Basal cortisol	18.3	18.4	4.6–22.80 *μ*g/dL
Sodium	151	137	132–146 mEq/L
Calcium	9.2	9.1	8.6–10.2 mg/dL
Creatinine	0.79	0.78	0.7–1.3 mg/dL
Potassium	5	4.6	3.5–5.5 mEq/L
Urea	26	28	19–48 mg/dL
Albumin	4.1	3.9	3.2–4.8 mg/dL
Osmolality	315	292	285–300
Urine			
Volume (1 day)	9.2	2.1	lt
Specific gravity	1.001	1.018	1.010–1.020
Osmolality (mosm/kg)	78	450	300–1090 mosm/kg
